# MDSi: Multi-omics Database for *Setaria italica*

**DOI:** 10.1186/s12870-023-04238-3

**Published:** 2023-04-27

**Authors:** Xukai Li, Siyu Hou, Mengmeng Feng, Rui Xia, Jiawei Li, Sha Tang, Yuanhuai Han, Jianhua Gao, Xingchun Wang

**Affiliations:** 1 Hou Ji Laboratory in Shanxi Province, Shanxi Agricultural University, Taiyuan, Shanxi 030031 China; 2 College of Life Sciences, Shanxi Agricultural University, Taigu, Shanxi 030801 China; 3 College of Agriculture, Shanxi Agricultural University, Taigu, Shanxi 030801 China; 4 South China Agricultural University, Guangzhou, Guangdong 510640 China; 5 Institute of Crop Sciences, Chinese Academy of Agricultural Sciences, Beijing, 100081 China

**Keywords:** Database, Multi-omics, Foxtail millet, *Setaria italica*, Tools

## Abstract

**Background:**

Foxtail millet (*Setaria italica*) harbors the small diploid genome (~ 450 Mb) and shows the high inbreeding rate and close relationship to several major foods, feed, fuel and bioenergy grasses. Previously, we created a mini foxtail millet, *xiaomi*, with an Arabidopsis-like life cycle. The de novo assembled genome data with high-quality and an efficient *Agrobacterium*-mediated genetic transformation system made *xiaomi* an ideal C_4_ model system. The mini foxtail millet has been widely shared in the research community and as a result there is a growing need for a user-friendly portal and intuitive interface to perform exploratory analysis of the data.

**Results:**

Here, we built a Multi-omics Database for *Setaria italica* (MDSi, http://sky.sxau.edu.cn/MDSi.htm), that contains *xiaomi* genome of 161,844 annotations, 34,436 protein-coding genes and their expression information in 29 different tissues of *xiaomi* (6) and JG21 (23) samples that can be showed as an Electronic Fluorescent Pictograph (xEFP) in-situ. Moreover, the whole-genome resequencing (WGS) data of 398 germplasms, including 360 foxtail millets and 38 green foxtails and the corresponding metabolic data were available in MDSi. The SNPs and Indels of these germplasms were called in advance and can be searched and compared in an interactive manner. Common tools including BLAST, GBrowse, JBrowse, map viewer, and data downloads were implemented in MDSi.

**Conclusion:**

The MDSi constructed in this study integrated and visualized data from three levels of genomics, transcriptomics and metabolomics, and also provides information on the variation of hundreds of germplasm resources that can satisfies the mainstream requirements and supports the corresponding research community.

**Supplementary Information:**

The online version contains supplementary material available at 10.1186/s12870-023-04238-3.

## Introduction

Foxtail millet (*Setaria italica*) (2n = 2x = 18) is thought to have been domesticated from the wild species green foxtail (*Setaria viridis*) more than 11,000 years ago in northern China [1]. With excellent drought tolerance and water-use efficiency, foxtail millet is still widely cultivated in the arid and semiarid regions in the world, particularly in China and India [2]. Foxtail millet are rich in essential amino acids, fatty acids and minerals, and easily digestible without allergenic potential [3]. It is being developed as a novel model system due to the advantages over other C_4_ plants, including small diploid genome (~ 450 Mb), high inbreeding rate and close relationship to several major foods, feed, fuel and bioenergy grasses [4–7].

Due to the rapid progress of high-throughput techniques (including genotyping by the array, RNA sequencing, metabolite identification, and phenotypic measurement), there has been rapid growth in related foxtail millet multidimensional omics data, and obtaining a high-quality genome assembly is no longer a bottleneck for most species. Even some species have multiple sets of genomes, such as rice (RAP-DB, https://rapdb.dna.affrc.go.jp; RGAP7, http://rice.uga.edu; RPAN, https://cgm.sjtu.edu.cn/3kricedb; IC4R, http://ic4r.org; RiceVarMap v2.0, http://ricevarmap.ncpgr.cn; RGI, https://riceome.hzau.edu.cn), and maize (MaizeGDB, https://maizegdb.org; MGR, http://maize.uga.edu; ZEAMAP, http://www.zeamap.com; MaizeMine, https://maizemine.rnet.missouri.edu; Gramene, https://www.gramene.org). A model crop system requires support of sufficient multi-omics data, such as genomics, RNA-seq and metabolic data. At present, the genome of four foxtail millet elite varieties or mutant, including Yugu1 [8], Zhanggu [9], TT8 [10], *xiaomi* [11] and Huagu11 [12] were assembled individually. The genome data of Yugu1 can be accessed in Phytozome [13], the data of Zhanggu was uploaded in BGI Foxtail millet Database (http://foxtailmillet.genomics.org.cn) whereas the information of Huagu11 was stored in China National GeneBank DataBase (CNGBdb) (https://db.cngb.org/search/project/CNP0000993/). Previously, we assembled the genome of the *xiaomi*, a mutant with a short life cycle like *Arabidopsis thaliana* of elite variety Jingu21 (JG21), to the scale of full chromosomes using single-molecule real-time sequencing (SMRT) and high-throughput chromosome conformation capture (Hi-C) [11]. We have also generated other types of omics data including the transcriptomic profiles of different tissues of *xiaomi* and its mutagenic parent JG21, the gene co-expression networks, as well as the single-nucleotide polymorphisms (SNPs) and insertion-deletion polymorphisms (InDels) of 398 foxtail millet and green foxtail germplasms and so on. Recently, the metabolomes of these germplasms were detected and used to multi-omics analysis [14]. An Arabidopsis-like life of *xiaomi* along with the plenty of multi-omics data greatly facilitate the application of *xiaomi*, as well as the foxtail millet in the C_4_ model system. A transversion from G to T occurred in the second exon of the *PHYC* (*Si9g09200*) gene, leading to the creation of a stop codon. It is the causal factor behind the *xiaomi* mini phenotype [11]. At present, *xiaomi* has been directly shared more than 40 research groups from 32 departments.

Phytozome [13], (yugu1: https://phytozome.jgi.doe.gov/pz/portal.html#!info?alias=Org_Sitalica) and BGI Foxtail millet Database (zhanggu: http://foxtailmillet.genomics.org.cn) hold sections for the foxtail millet, in which the genome sequence of the yugu1 and zhanggu are retrievable. In addition, their genes ID are totally different, e.g. Seita.xGxxxxxx for yugu1 and Millet_GLEAN_xxxxxxxx for zhanggu, which greatly restrict the application of genome data. The lack of essential information in molecular breeding, such as phenotypic information, molecular markers, and gene expression data, impedes progress in the field. To overcome this challenge, there is an urgent need for a multi-dimensional database that encompasses multiple levels of omics data for the same panel. This comprehensive database should comprise high-quality data on genomic variations, reliable quantitation of gene expression, detailed measurement of cellular metabolites, and high-throughput characterization of morphological phenotypes for large, representative, and diverse populations. Furthermore, mapping results based on these data and generated using different methods will directly contribute to further scientific studies. The resource should also be equipped with a user-friendly mechanism for data retrieval, convenient visualization tools, and comprehensive annotation information.

Advances in high-throughput technologies have given rise to huge amounts of omics data for many species. However, wet-lab biologists prefer a user-friendly portal and intuitive interface to perform exploratory analysis of the data. The data of the model plants and major crops have been organized into the corresponding databases with user-friendly interface, including *A. thaliana* [15], rice (*Oriza sativa*) [16–18], maize (*Zea mays*) [19, 20], soybean (*Glycine max*) [21], sorghum (*Sorghum bicolor*) [22] and cotton (*Gossypium* sp.) [23, 24], etc. To facilitate the cross-species comparison, several comprehensive databases have been constructed, such as Phytozome [13], PlantGDB [25], Gramene [26]. Although these comprehensive databases already encompass the foxtail millet genomic data (Yugu1) [8], a dedicated database including the information of different varieties along with flexible analysis tools, are not yet available.

In this study, the MDSi (Multi-omics Database for *Setaria italica*, http://sky.sxau.edu.cn/MDSi.htm) was developed that contains two annotated genomes (*xiaomi* and Yugu1), the expression atlas from various tissues of *xiaomi* and JG21, transcription factors, transposons and NcRNAs datasets of *xiaomi*, molecular markers (SNPs and InDels) among 398 foxtail millet and green foxtail germplasms, as well as their metabolite traits. The database also provides intuitive search portal and common analysis tools, such as Blast, GBrowe and JBrowse. The Electronic Fluorescent Pictograph (xEFP) browser (Yang, Zhang et al. 2020), which can display the gene-expression data in-situ, is also another feature of MDSi. This large dataset will allow association mapping that will advance understanding of the genetic architecture of specific phenotypes, which, in turn, will allow deep annotation of the foxtail millet genome. While genetic and genomic research is progressing in foxtail millet, the related database resources have lagged behind and are limited for next level analyses—functional genome studies such as QTL mapping.

## Results

### Gene search

**Fig.1:**
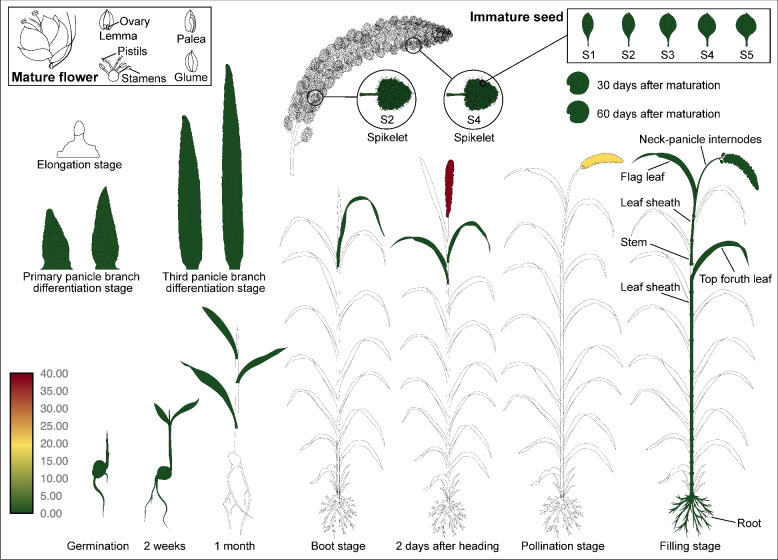
The visualization of the *Si1g01230* gene expression in different tissues

We built a MDSi (Multi-omics Database for *Setaria italica*, http://sky.sxau.edu.cn/MDSi.htm, Fig. S1), and the gene data of both *xiaomi* and Yugu1 were deployed in our database. The gene information of interest was able to be obtained either by Gene Search in Search menu or according to the corresponding annotation (Browse → Annotation) or known functions (Browse → Functional Category). At present, 161,844 annotations of 33,785 genes and 4,944 functional categories were collected depending on the databases of non-redundant proteins (Nr), Kyoto Encyclopedia of Genes and Genomes (KEGG) and Gene Ontology (GO), respectively. The predicted transcription factors and transposons were also classified in Browse menu, respectively. All the searching results guide users to the gene information (Gene info) page of the target gene (Fig.S2) in which several modules were integrated including Gene Structure (GBrowse, JBrowse), Gene Sequence, Gene Expression (the updated xEFP, Fig. 1), Protein Homologs and Gene Annotation.

### Annotation

The gene annotations (161,844) were listed as a table in the Annotation module in Browse menu (Fig.S3). The genes of interest can be screened in this module by the Gene ID or annotated keywords. The planteome ontology information of the corresponding gene was indicated in the last column linking the Planteome ontology module in Search menu. The predicted transcription factors and transposons were independently listed in corresponding modules in Browse menu. There were 1848 transcript factors belong to 55 classes and 394,434 transposon related sequences within 40 classes. Moreover, 1120 trait ontologies of foxtail millet were screened referred to the data in China rice data center (https://www.ricedata.cn/ontology/) that can be obtained in the Trait Ontology module in Browse menu or Trait Ontology Search module in Search menu.

### Marker search

**Fig.2:**
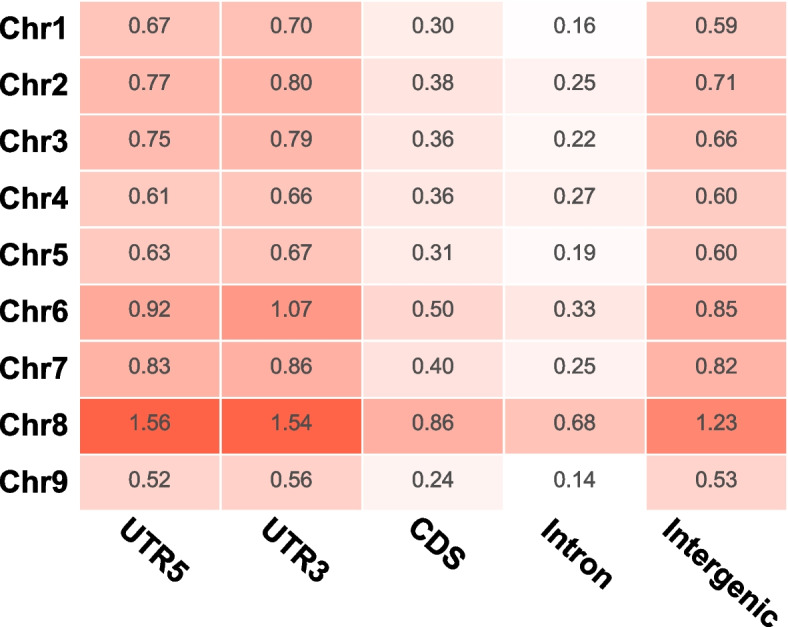
The variation frequency distribution of the SNPs and InDels

The 4,158,075 SNPs and InDels among 398 foxtail millet (360) and green foxtail (38) germplasms were collected and deployed. The information can be screened by region in chromosome, Gene ID or Variation ID in Variation Search page (Search menu, Fig.S4). JBrowse allows for the visualization of gene mutations and their impact on genes, including both synonymous, non-synonymous, stopgain, stoplost, startlost, splice, and so on (Tools → JBrowse, Fig.S5). Notably, the locations of the variations were also marked by the common features such as untranslated regions (UTR), exon and intron as so on. Additionally, the variation distributions of each germplasm were summarized in SNPs Variation and InDels Variation page (Browse menu, Fig.S6 and Fig.S7). The chromosome 8 shows highest variation frequency (the ratio of SNPs and InDels numbers to the length of corresponding region) in different regions including intron, CDS, UTR5, UTR3 and intergenic sequences (Fig. 2). Interestingly, the regions of CDS and intron take less variations, especially in latter sequences.

### Online tools

**Fig.3:**
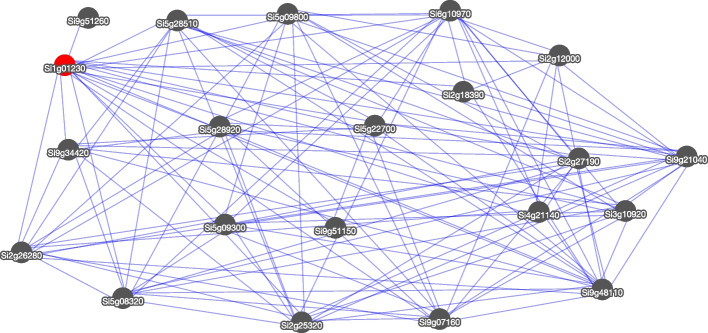
The gene network of *Si1g01230* gene

BLAST was implemented using ViroBlast [27] for identifying sequence homology in genomic scaffolds, coding sequences, or proteins levels. The Gbrowse [28] and Jbrowse [29] were included simultaneously for gene structure visualization. The MDSi also provided a special tool for displaying the network of a gene of interest (Gene network in Tools menu) based on the context likelihood of relatedness (CLR) algorithm. For instance, total 20 genes would be related to the trithorax group protein osa-like (Si1g01230) with the p-value more than 0.98 (Fig. 3 and Fig.S8). The expression level of the screened or other genes of interest can be visualized in Expression Visualization module of Tools menu (Fig.S9). The corresponding raw data were also provided for further analysis. Both the tool and the aforementioned xEFP provided users with intuitive graphs of the gene expression patterns in different tissues at multiple- or single-gene level, respectively. The Chromosome Map can visualize the location of these genes (Fig.S10). The SiTRAIT and SiMetabolite were used to display data. The SiTRAIT (Tools → SiTRAIT) contains 18 agronomic traits, such as plant height, thousand seed weight, tiller number, length and width of leaf, etc. And the metabolic data of grains were also available in SiMetabolite (Tools → SiMetabolite).

### Data download

The Download page (Fig.S11) was provided for users to obtain entire datasets as needed, including genomic scaffolds, predicted gene sequences and the corresponding proteins in the FASTA format. The germplasms information can also be collected directly here. Moreover, the download functions also distributed in some modules described above if needed.

## Discussion

Foxtail millet is being developed as a novel C_4_ model which demands the high-throughput multi-omics data. At present, there were 5 de novo assembled genomic data of 4 elite cultivars [8–10, 12] and a mutant *xiaomi* with shorter life cycle [11]. We also published the RNA-seq data of 11 tissues of *xiaomi* at different stages [11]. Additionally, twenty-three RNA-seq data of the elite varieties JG21, a benchmark for high-quality foxtail millet [30] were also prepared. The whole-genome resequencing data of 398 germplasms, including 360 foxtail millets and 38 green foxtails, were reported for reveal genomic regions associated with many traits including 2557 metabolites identified by UPLC–MS/MS [14]. Recently, the restriction site associated DNA sequencing (RAD sequencing) data of a total of 1127 foxtail millet cultivars were published [31]. Therefore, the construction of a flexible and dedicated multi-omics database for foxtail millet is therefore an urgent priority.

At present, there is still no dedicated bioinformatics database for foxtail millet. The genome data of Yugu1 were implemented in Phytozome that provides a remarkably user-friendly interface for the cross-species comparison and analysis among multiple plants. However, for the time being, Phytozome does not provide in-depth and multidimensional species-specific data and analysis tools. Therefore, many dedicated databases for the important plants were constructed independently. FmMDb (Foxtail millet Marker Database) is the first database that organized and visualized large-scale marker datasets of Foxtail millet [32, 33], but unfortunately, it is no longer accessible. SIFGD (*S. italica* Functional Genomics Database) [34] was established for bioinformatics analyses of gene function or regulatory modules using integrated the genome sequences of Yugu1 [8] and Zhanggu [9]. To this date, SIFGD remains an important foxtail millet genomics database. However, it is clear that, due to the progress of research at the time, it lacks relevant information at the transcriptional and metabolic levels.

The construction of a dedicated database for foxtail millet is necessary to facilitate effective analysis and utilization of the available genomic datasets. Such a database could provide comprehensive and multidimensional species-specific data, as well as sophisticated analysis tools, thereby promoting better understanding of the plant's molecular biology and accelerating the development of novel genomics-assisted breeding strategies. In comparison to the currently published foxtail millet genomes, *xiaomi* has been found to be the most complete. Similarly, when compared to other available foxtail millet genome databases, our MDSi ranks as the most comprehensive and reliable (Table 1).

**Table 1:** Comparison among the database of *xiaomi*, TT8, Yugu1, and Zhanggu

Statistic	*xiaomi*	Yugu1	Zhanggu	TT8
Estimated size (Mb)	438	510	485	NA
Assembly size (Mb)	429.9	405.7	423.4	477.6
Genome coverage	98%	80%	~ 86%	97%
Contigs	419	6,791	71,571	NA
Contig N50 (kb)	18,838.5	126.3	25.4	NA
Scaffolds	366	336	37,727	2,688
Scaffold N50 (Mb)	42.4	47.3	1.0	53.2
Number of gaps	48	6,455	34,282	7,136
Number of N’s (bp)	NA	4,826,887	26,194,380	3,381,668
Genome wide annotation	org.Si.eg.db (R package)	NA	NA	NA
Database data sets	Genome, Transcriptome, Metabolome, Variations	Genome	Genome	NA
Database search	Gene, Variation, Planteome Ontologies, Trait Ontology	Keyword	Error: Access denied	NA
Database tools	Blast, Gene network, Jbrowse, Motif, Chromosome Map, SiTRAIT, SiMetabolite	Blast, Jbrowse, Motif, Synteny, GSEA	Error: Access denied	NA
Genetic transformation	Yes. The average transformation efficiency for NPTII is 23.28%	NA	NA	NA

## Conclusion

MDSi constructed in this study follows the frontiers of cereal is the only multi-omics database that is currently operational and maintained. The MDSi constructed in this study integrated and visualized data from three levels of genomics, transcriptomics and metabolomics, and also provides information on the variation of hundreds of germplasm resources that can satisfies the mainstream requirements and supports the corresponding research community.

## Materials and methods

### Plant materials

*xiaomi* was identified from an EMS-mutagenized M2 population of Jingu21, a variety of foxtail millet widely cultivated in North China for its good grain quality and high yield. The *xiaomi* mutant shows short life-cycle like *A. thaliana*. The *xiaomi* mutant was maintained by self-pollination in the laboratory for ten generations, leading to a very low level of heterozygosity. A total of 398 accessions, including 360 foxtail millets and 38 *S. viridis*, were also used in this study [14].

### Architecture and implementation of MDSi

**Fig.4:**
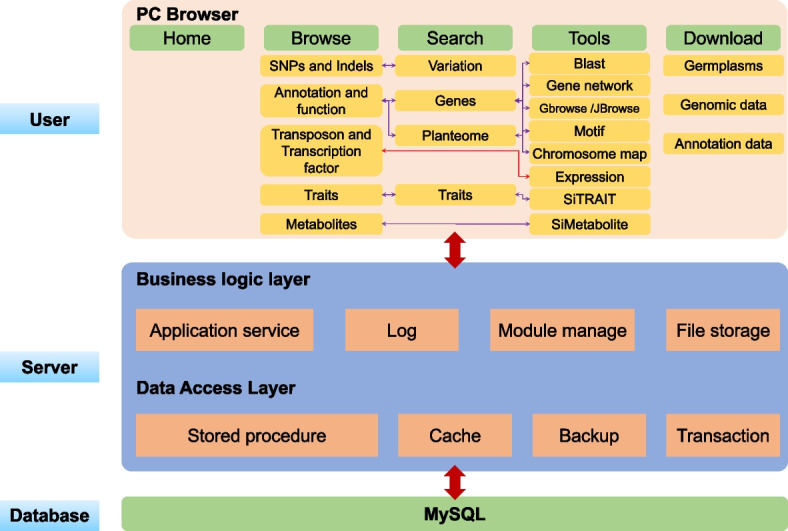
The schema architecture of MDSi

The MDSi was implemented in the linux system (CentOS 7), including PHP, JAVA, Apache web server, MySQL database management, and Perl CGI. An interactive Web interface was designed for users (Fig.S1). User query information is transferred into PHP script and explained by corresponding interpreter invoked by apache web server (Fig. 4). The MDSi comprises a set of relational databases that store processed data in MySQL. To facilitate effortless access to the MDSi for basic research applications or biological analysis, an interactive web interface has been created. The interface utilizes PHP script to rapidly transmit user query information and extract data from the MySQL database management system to generate report pages. Additionally, the genome visualization tool has been integrated using the genetic genome browser, comprising Gbrowse [28] and Jbrowse [29], which provides improved performance and interface enhancements, including a robust faceted track selector. For interactive alignment of genome sequences, BLAST was performed by ViroBLAST, an independent web server for flexible queries of similar nucleotide and amino acid sequences. The browser finally displays the data by graphical manner.

### Genomic data of xiaomi

We assembled reference-grade genome sequence (~ 429.9 Mb) de novo previously using three approaches, including the short read paired-end sequencing (Illumina HiSeq X Ten), the single molecule real-time (SMRT) sequencing (PacBio RSII and Sequel) and the chromosome conformation capture sequencing (Hi-C), for promoting its application in C_4_ model system [11]. Compared with the previous Yugu1 [8] genone (∼400.9 Mb) (9), Zhanggu [9] genome (∼423.4 Mb), the *xiaomi* genome in MDSi is a high-quality, chromosome-level assembly that is more intact. The data were the core foundation of the MDSi.

### Genomic data of germplasms

The genome of Yugu1 (∼400.9 Mb), which was well visualized in Phytozome, was also added to the MDSI. Moreover, the MDSi also provides the whole-genome resequencing (WGS) results of 398 germplasms, including 360 foxtail millets and 38 green foxtails with an average of depth of 10 times [14]. The paired-end resequencing reads were mapped to the xiaomi reference genome with BWA using the default parameters. The SAMtools, Picard package and Genome Analysis Toolkit (GATK) and VCFtools were performed to filter duplicated reads, detect SNPs and filter data (missing data < 0.9, minor allele frequency ≥ 0.05 and depth ≥ 5). We identified 3,826,381 SNPs and 331,694 indels.

### Transcriptomics data

To quantify the expression of known genes, we measured 29 RNA-seq in diverse tissues representing the major organ systems and varying developmental stages of xiaomi and JG21 with three biological repeats. RNA-seq data of 29 different tissues of *xiaomi* (6) and JG21 (23) including germinated-seed, seedling, leaf, leaf sheath, leaf-veins, mesophyll, internode, root, panicle, immature spikelet and seed at different stages or positions are encompassed in MDSi. The clean reads were obtained using Trimmomatic (0.38), and were subsequently aligned to *xiaomi* reference genomes using HISAT2 (v2.0.5). The Transcripts Per Kilobase per Million mapped reads (TPM) of the annotated genes were normalized using R with default parameters. A total of 25,949 informative genes with TPM more than 10 in at least one tissue were obtained and gene pairs with the absolute similarity of expression correlation > 0.90 were used as the final dataset. On each gene page, tools for viewing temporal and spatial expression data are now embedded in the Expression table and render as soon as the page is selected. Mouse-overs display the read counts for individual data points. The values of genes expression transform set to log2 to provide a more direct comparison.

### Metabolic data

The metabolic data of grains of 398 germplasms obtained by ultra-performance liquid chromatography-tandem mass spectrometry (UPLC–MS/MS) were also available in the MDSi [14] (Browse → Metabolic Data).

## Supplementary Information

**Additional file 1: Fig. S1.** The home page of MDSi. **Fig. S2.** The gene info page of MDSi. **Fig. S3. **The annotation page of MDSi. **Fig. S4.** The variation search page of MDSi. **Fig. S5.** The JBrowse shows the gene mutations and their impact on Si9g49990 genes. **Fig. S6.** The SNP variation page of MDSi. **Fig. S7.** The InDel variation page of MDSi. **Fig. S8.** The network result page of Si1g01230 gene. **Fig. S9.** The network result of Si1g01230. **Fig. S10.** The chromosome location of genes related to Si1g01230. **Fig. S11.** The download page of MDSi.

## Data Availability

The genome assembly and annotation of *xiaomi* are also available at Genome Warehouse in the Beijing Institute of Genomics Data Center (https://bigd.bigac.cn/) under accession number GWHAAZD00000000. The raw sequence data have been deposited in the Beijing Institute of Genomics Data Center with the following accession numbers: CRA001973 (Genome sequencing of *xiaomi* by PacBio), CRA001968 (Hi-C of *xiaomi*), CRA001972 (isoform sequencing of *xiaomi*), CRA001953 (RNA-seq of *xiaomi* tissues), CRA001954 (RNA-seq of Jingu21), CRA001974 (non-coding RNAs), and CRA002636 (genome re- sequencing of 398 accessions). The metabolomics data have been deposited on the UCSD Metabolomics Workbench under the accession no. ST002168. The MDSi can be freely accessed at http://sky.sxau.edu.cn/MDSi.htm via the World Wide Web. A reliable data management system has been developed and all newly released information will be updated on this website. Inquiries concerning the database should be directed by email to xukai_li@sxau.edu.cn.
