# Supplementary material for: MDSi: Multi-omics Database for Setaria italica
Source: BMC Plant Biol. 2023 Apr 27;23:223. doi: 10.1186/s12870-023-04238-3 (PMC10134609; doi:10.1186/s12870-023-04238-3)
Supplement: Supplementary file 1 — Additional file 1: Fig. S1. The home page of MDSi. Fig. S2. The gene info page of MDSi. Fig. S3. The annotation page of MDSi. Fig. S4. The variation search page of MDSi. Fig. S5. The JBrowse shows the gene mutations and their impact on Si9g49990 genes. Fig. S6. The SNP variation page of MDSi. Fig. S7. The InDel variation page of MDSi. Fig. S8. The network result page of Si1g01230 gene. Fig. S9. The network result of Si1g01230. Fig. S10. The chromosome location of genes related to Si1g01230. Fig. S11. The download page of MDSi. [file 12870_2023_4238_MOESM1_ESM.pdf]

MDSi: Multi-omics Database for *Setaria italica*

Xukai Li[1,2,†], Siyu Hou[1,3,†], Mengmeng Feng[2], Rui Xia[4], Jiawei Li[4], Sha Tang[5], Yuanhuai Han[1,3], Jianhua Gao[1,2,*], Xingchun Wang[1,2,*]

[1] *Hou Ji Laboratory in Shanxi Province, Shanxi Agricultural University, Shanxi, Taiyuan, 030031, China*

[2] *College of Life Sciences, Shanxi Agricultural University, Shanxi, Taigu, 030801, China*

[3] *College of Agriculture, Shanxi Agricultural University, Shanxi, Taigu, 030801, China*

[4] *South China Agricultural University, Guangzhou, Guangdong 510640, China*

[5] *Institute of Crop Sciences, Chinese Academy of Agricultural Sciences, Beijing, 100081, China.*

[†] Contributed equally to this work

* Correspondence to Jianhua Gao (jhgao@sxau.edu.cn) or Xingchun Wang (wxingchun@sxau.edu.cn).

Supplementary Information

Additional file 1:

Fig.S1 The home page of MDSi.

Fig.S2 The gene info page of MDSi.

Fig.S3 The annotation page of MDSi.

Fig.S4 The variation search page of MDSi.

Fig.S5 The JBrowse shows the gene mutations and their impact on *Si9g49990* genes.

Fig.S6 The SNP variation page of MDSi.

Fig.S7 The InDel variation page of MDSi.

Fig.S8 The network result page of *Si1g01230* gene.

Fig.S9 The network result of *Si1g01230*.

Fig.S10 The chromosome location of genes related to Si1g01230.

Fig.S11 The download page of MDSi.

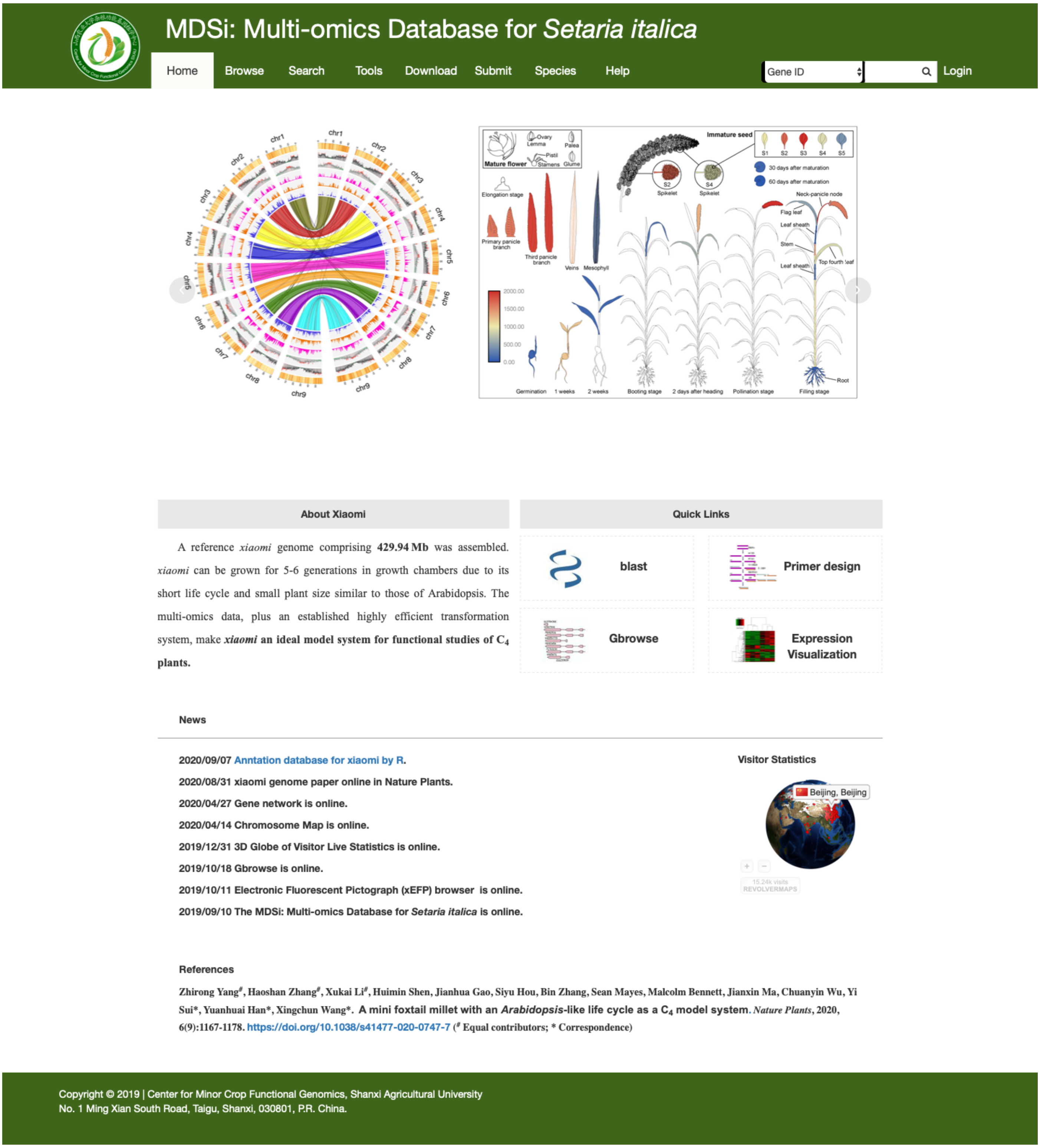

Fig.S1 The home page of MDSi.

Gene Info

Gene Name: Si1g01010.1 **Location:** 1:32610..33311 (+) **Variation:** SNP Indel

Description: ATP synthase CF0 A subunit [Zea mays]

Gene Structure

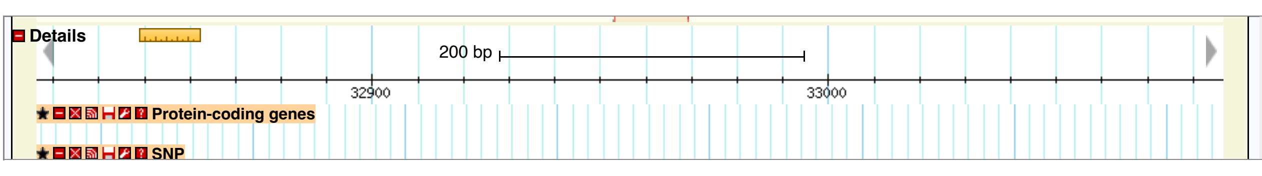

Gene Sequence

Please select the file Download

Genomic sequence [702]

Key: 3'UTR CDS 5'UTR

Show flanking sequence: upstream: 0 downstream: 0 Submit

>Si1g01010.1

ATGAATATTACACCATGTTCCATTAAAACACTCAAGGGGTTATATGATATATCGGGTGTAGAAGTAGGCCAACACTTCTATTGGCAAATAGGAGGTTTTCAAATTCATGCCCAAGTACTCATCAC
TTCTTGGGTCGTAATTACTATTTTGCTAGGTTCAGTTATCATAGCTGTTCGGAATCCACAAACTATCCCGACCGACGGTCAGAATTTCTTCGAATATGTCCTTGAGTTTATTCGAGACTTGAGC
AAAACTCAGATTGGAGAAGAATATGGTCCCTGGGTTCCCTTTATTGGAACTATGTTCCTTTTTATTTTTGTTTCGAATTGGTCAGGTGCTCTTTTACCTTGGAAAATTATAGAGTTACCTCATGG
AGAATTAGCAGCGCCCACGAATGATATAAATACTACTGTTGCTTTAGCTTTACTAACATCAGCGGCATATTTTTATGCGGGTCTTAGCAAAAAAGGATTGAGTTATTTCGAGAAATATATTAAACC
AACCCCAATCCTTTTACCAATTAACATCCTAGAAGATTTCACAAAACCATTATCGCTTAGTTTTCGACTTTTCGGAAATATATTGGCGGATGAATTAGTCGTTGTTGTTCTTGTTTCTTTAGTCCC

CDS sequence [702]

Transcript sequence [702]

Peptide sequence [233]

Gene Expression

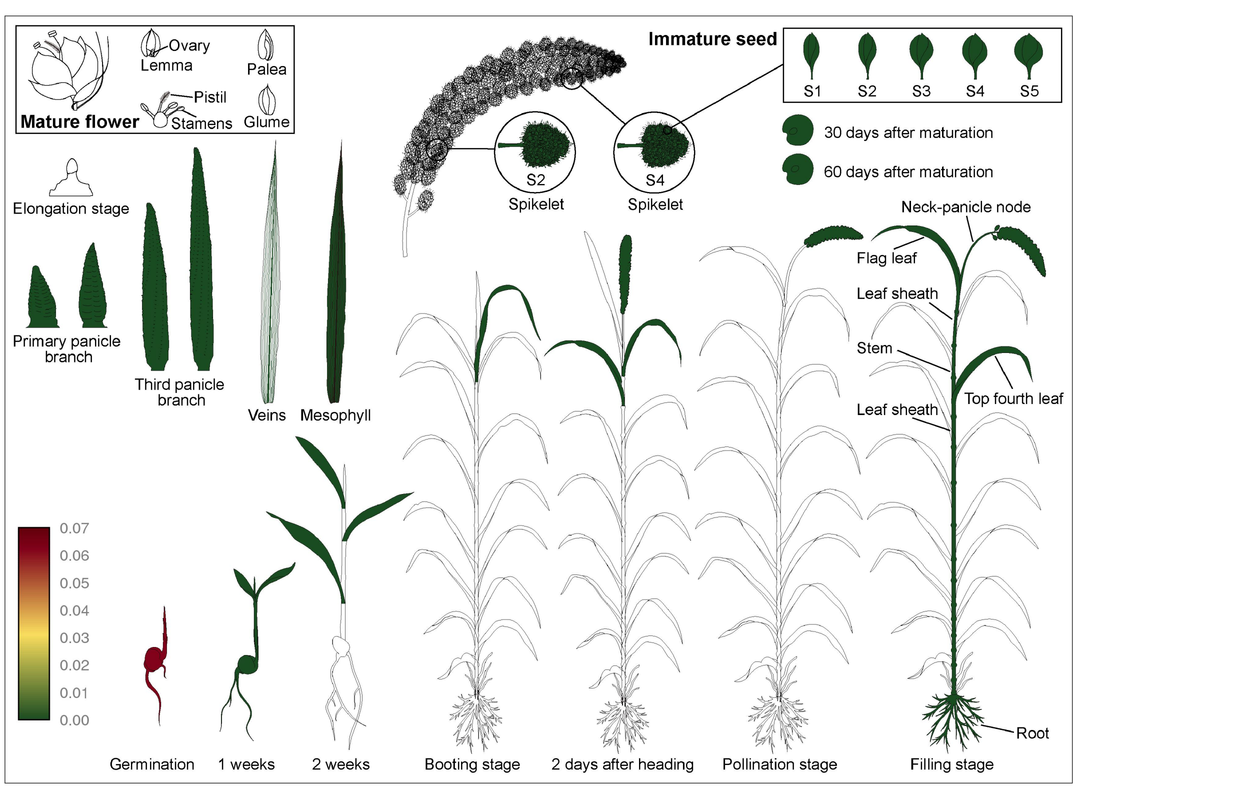

Protein Homologs

Download All

Organism	Transcript Name	Identity (%)	Alignment length	E value	Score
Athaliana_447_Araport11	ATCG00150.1	90.1	232	7.6e-117	417.9
Athaliana_447_Araport11	ATCG00150.1	90.1	232	7.6e-117	417.9
BdistachyonABR2_337_v1	Brdisv1ABR21046924m.p	95.2	207	5.2e-112	401.7
BdistachyonABR2_337_v1	Brdisv1ABR21000892m.p	97.9	146	8.2e-81	298.1
Taestivum_296_v2.2	Traes_2DL_585424A8F.1	98.7	223	1.8e-122	438.7
Taestivum_296_v2.2	Traes_5DS_646258D1D.1	98.6	145	6.4e-80	297.4
Taestivum_296_v2.2	Traes_3AS_FD5003ACF.1	97.8	138	4.3e-76	284.6
Taestivum_296_v2.2	Traes_7AS_0EF508307.1	98.5	131	1.7e-72	272.7
Taestivum_296_v2.2	Traes_4AL_0EF5083071.1	98.5	131	1.7e-72	272.7
Taestivum_296_v2.2	Traes_6BL_0EF508307.1	98.5	131	1.7e-72	272.7
Taestivum_296_v2.2	Traes_2BL_0EF508307.1	98.5	131	1.7e-72	272.7
Taestivum_296_v2.2	Traes_1BL_0EF508307.1	98.5	131	1.7e-72	272.7
Taestivum_296_v2.2	Traes_5DS_E6273D645.1	98.5	131	1.7e-72	272.7
Taestivum_296_v2.2	Traes_2AL_0EF508307.1	98.5	131	1.7e-72	272.7
Taestivum_296_v2.2	Traes_6BS_0EF508307.1	98.5	131	1.7e-72	272.7
Taestivum_296_v2.2	Traes_7AL_0EF508307.1	98.5	131	1.7e-72	272.7
Taestivum_296_v2.2	Traes_5AL_0EF508307.1	98.5	131	1.7e-72	272.7
Taestivum_296_v2.2	Traes_6DS_0EF508307.1	98.5	131	1.7e-72	272.7
Taestivum_296_v2.2	Traes_6AS_0EF508307.1	98.5	131	1.7e-72	272.7
Taestivum_296_v2.2	Traes_3B_0EF508307.1	98.5	131	1.7e-72	272.7
Taestivum_296_v2.2	Traes_7BL_D041399F0.1	98.5	131	1.7e-72	272.7
Taestivum_296_v2.2	Traes_2BS_0EF508307.1	98.5	131	1.7e-72	272.7
Taestivum_296_v2.2	Traes_3DL_0EF508307.1	98.5	131	1.7e-72	272.7
Taestivum_296_v2.2	Traes_2DS_0EF508307.1	98.5	131	1.7e-72	272.7
Taestivum_296_v2.2	Traes_4DS_0EF508307.1	98.5	131	1.7e-72	272.7
Taestivum_296_v2.2	Traes_5AS_0EF508307.1	98.5	131	1.7e-72	272.7
Taestivum_296_v2.2	Traes_4BL_0EF508307.1	98.5	131	1.7e-72	272.7
Taestivum_296_v2.2	Traes_7DL_0EF508307.1	98.5	131	1.7e-72	272.7
Taestivum_296_v2.2	Traes_1AL_0EF508307.1	98.5	131	1.7e-72	272.7
Xiaomi	Si1g01010.1	100.0	233	2.7e-129	459.1
Xiaomi	Si0g13890.1	99.6	233	8.0e-129	457.6
Xiaomi	Si0g01320.1	100.0	230	1.2e-127	453.8
Xiaomi	Si0g01730.1	100.0	230	1.2e-127	453.8
Xiaomi	Si0g02910.1	100.0	230	1.2e-127	453.8
Xiaomi	Si0g03590.1	100.0	230	1.2e-127	453.8
Xiaomi	Si0g03950.1	100.0	230	1.2e-127	453.8
Xiaomi	Si0g05050.1	100.0	230	1.2e-127	453.8
Xiaomi	Si0g06160.1	100.0	230	1.2e-127	453.8
Xiaomi	Si0g11900.1	100.0	230	1.2e-127	453.8
Xiaomi	Si0g12500.1	100.0	230	1.2e-127	453.8
Xiaomi	Si0g13090.1	100.0	230	1.2e-127	453.8
Xiaomi	Si0g14320.1	100.0	230	1.2e-127	453.8
Xiaomi	Si0g14620.1	100.0	230	1.2e-127	453.8
Xiaomi	Si0g14830.1	100.0	230	1.2e-127	453.8
Xiaomi	Si0g15690.1	100.0	230	1.2e-127	453.8
Zmays_493_RefGen_V4	GRMZM5G875287_P01	100.0	230	3.8e-127	453.8
FtChromosomeV2.IGDBv2	FtPinG0001790400.01.T01	63.8	105	6.7e-30	128.3
Ghirsutum_527_v2.1	Gohir.A05G086000.2.p	92.5	146	1.8e-75	281.6
Ghirsutum_527_v2.1	Gohir.A03G085501.1.p	86.1	144	1.2e-68	258.8
Ghirsutum_527_v2.1	Gohir.D08G103720.1.p	65.5	174	7.8e-55	213.0
Ghirsutum_527_v2.1	Gohir.A10G169433.1.p	91.3	92	1.9e-45	181.8
Ghirsutum_527_v2.1	Gohir.D07G034200.2.p	67.1	146	5.6e-45	180.3
Ghirsutum_527_v2.1	Gohir.D02G023550.1.p	82.0	100	3.6e-36	151.0
Ghirsutum_527_v2.1	Gohir.D02G093150.1.p	88.1	84	4.6e-31	134.0
Ghirsutum_527_v2.1	Gohir.D09G014950.1.p	78.1	73	5.1e-22	104.0
Hvulgare_462_r1	HORVU6Hr1G049150.1	98.7	230	4.5e-126	450.7
Hvulgare_462_r1	HORVU0Hr1G024810.1	98.9	181	1.7e-101	369.0
Hvulgare_462_r1	HORVU0Hr1G028530.1	98.8	161	2.0e-89	328.9
Hvulgare_462_r1	HORVU0Hr1G033900.1	98.8	161	2.0e-89	328.9
Hvulgare_462_r1	HORVU0Hr1G036790.1	98.8	161	2.0e-89	328.9
Hvulgare_462_r1	HORVU0Hr1G032740.1	98.8	161	2.0e-89	328.9
Hvulgare_462_r1	HORVU0Hr1G024480.1	98.8	161	2.0e-89	328.9
Hvulgare_462_r1	HORVU4Hr1G045380.6	99.4	156	1.2e-81	303.1
Hvulgare_462_r1	HORVU5Hr1G013580.2	89.1	137	5.2e-66	251.1
Hvulgare_462_r1	HORVU4Hr1G045380.5	99.2	130	7.6e-65	247.3
Hvulgare_462_r1	HORVU0Hr1G024810.2	81.0	121	2.8e-51	202.2
Hvulgare_462_r1	HORVU6Hr1G049150.2	81.0	121	2.8e-51	202.2
Hvulgare_462_r1	HORVU4Hr1G045380.4	100.0	87	5.1e-45	181.4
Hvulgare_462_r1	HORVU7Hr1G005040.7	100.0	75	6.7e-37	154.5
Hvulgare_462_r1	HORVU7Hr1G005040.2	100.0	75	6.7e-37	154.5
Hvulgare_462_r1	HORVU4Hr1G045350.1	97.3	73	1.6e-35	149.8
Hvulgare_462_r1	HORVU2Hr1G006240.1	91.9	74	1.2e-33	143.7
Hvulgare_462_r1	HORVU5Hr1G013580.1	100.0	61	2.5e-28	125.9
Hvulgare_462_r1	HORVU4Hr1G045380.7	97.9	47	2.3e-21	102.8
Hvulgare_462_r1	HORVU5Hr1G013580.3	73.8	61	3.0e-21	102.4
Hvulgare_462_r1	HORVU5Hr1G067560.1	98.1	53	6.3e-19	94.7
Hvulgare_462_r1	HORVU7Hr1G005040.3	100.0	38	1.8e-13	76.6
Hvulgare_462_r1	HORVU7Hr1G005040.4	100.0	36	1.1e-12	73.9
Hvulgare_462_r1	HORVU4Hr1G045380.3	100.0	20	8.8e-05	47.8
Sbicolor_454_v3.1.1	Sobic.003G169200.1.p	99.6	230	4.4e-127	451.8
Sbicolor_454_v3.1.1	Sobic.003G142900.1.p	94.4	126	2.5e-66	250.0
Sitalica_312_v2.2	Seita.J019300.1.p	100.0	85	4.6e-46	182.6
Sitalica_312_v2.2	Seita.6G110000.1.p	83.6	61	2.0e-17	87.4
Sviridis_500_v2.1	Sevir.6G120200.1.p	85.2	61	1.6e-18	91.3

Gene Annotation

Download All

ID	Type	Description
gi|11467186|ref|NP_043019.1	Nr	ATP synthase CF0 A subunit [Zea mays]
GO:0046933	GO	proton-transporting ATP synthase activity, rotational mechanism
GO:0016021	GO	integral component of membrane
GO:0042777	GO	plasma membrane ATP synthesis coupled proton transport
GO:0045263	GO	proton-transporting ATP synthase complex, coupling factor F(o)
GO:0009535	GO	chloroplast thylakoid membrane
GO:0005886	GO	plasma membrane
K02108	KEGG	K02108 F-type H+-transporting ATPase subunit a

Publications

Author/Title	Source	Associated loci	Data
No Data			

Copyright © 2019 | Center for Minor Crop Functional Genomics, Shanxi Agricultural University
No. 1 Ming Xian South Road, Taigu, Shanxi, 030801, P.R. China.

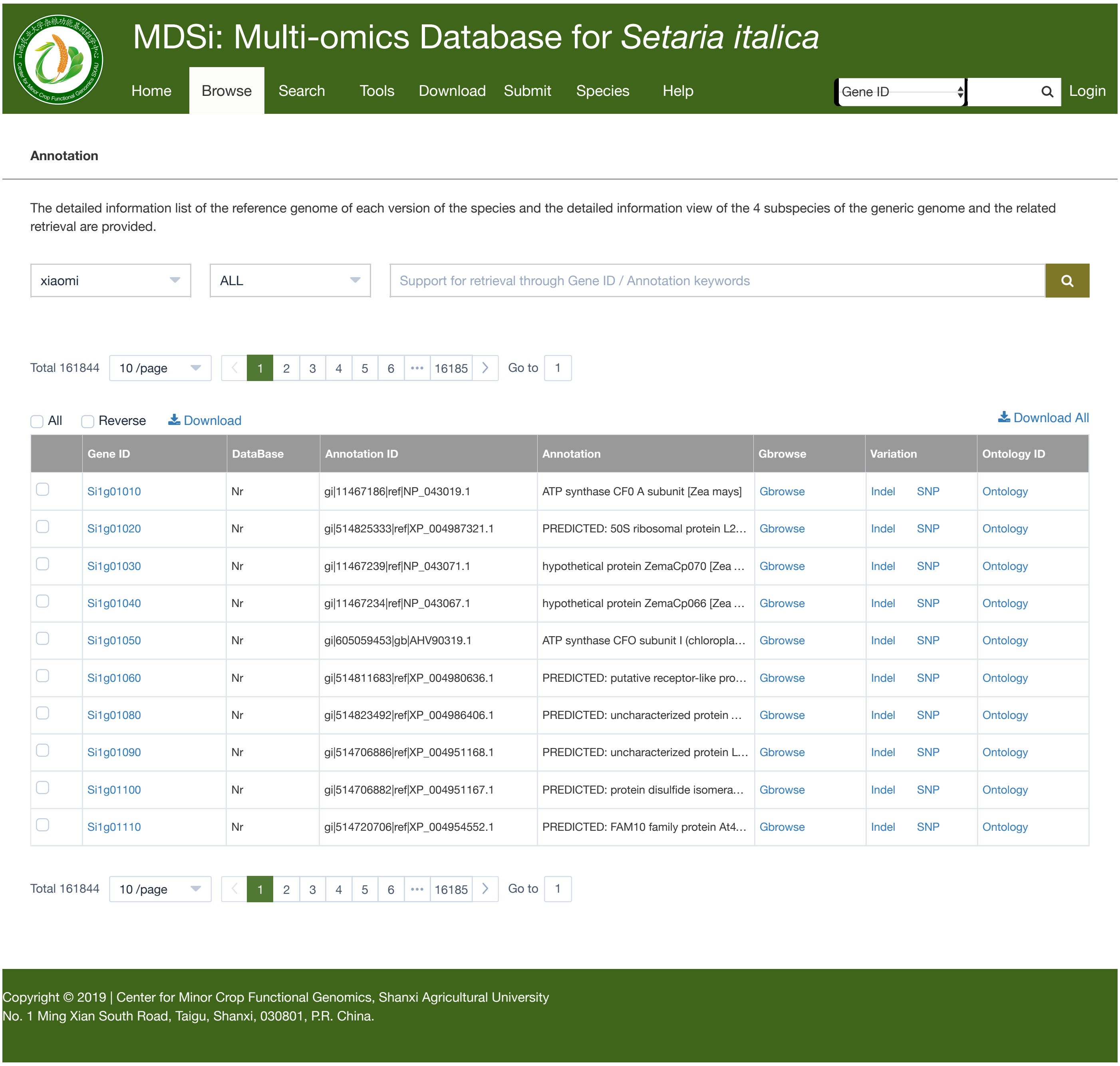

MDSi: Multi-omics Database for *Setaria italica*

Home Browse Search Tools Download Submit Species Help | Gene ID | Login

Annotation

The detailed information list of the reference genome of each version of the species and the detailed information view of the 4 subspecies of the generic genome and the related retrieval are provided.

xiaomi | ALL | Support for retrieval through Gene ID / Annotation keywords

Total 161844 | 10 /page | 1 2 3 4 5 6 ••• 16185 | Go to 1

All | Reverse | Download | Download All

	Gene ID	DataBase	Annotation ID	Annotation	Gbrowse	Variation	Ontology ID
☐	Si1g01010	Nr	gi|11467186|ref|NP_043019.1	ATP synthase CF0 A subunit [Zea mays]	Gbrowse	Indel SNP	Ontology
☐	Si1g01020	Nr	gi|514825333|ref|XP_004987321.1	PREDICTED: 50S ribosomal protein L2…	Gbrowse	Indel SNP	Ontology
☐	Si1g01030	Nr	gi|11467239|ref|NP_043071.1	hypothetical protein ZemaCp070 [Zea …	Gbrowse	Indel SNP	Ontology
☐	Si1g01040	Nr	gi|11467234|ref|NP_043067.1	hypothetical protein ZemaCp066 [Zea …	Gbrowse	Indel SNP	Ontology
☐	Si1g01050	Nr	gi|605059453|gb|AHV90319.1	ATP synthase CFO subunit I (chloropla…	Gbrowse	Indel SNP	Ontology
☐	Si1g01060	Nr	gi|514811683|ref|XP_004980636.1	PREDICTED: putative receptor-like pro…	Gbrowse	Indel SNP	Ontology
☐	Si1g01080	Nr	gi|514823492|ref|XP_004986406.1	PREDICTED: uncharacterized protein …	Gbrowse	Indel SNP	Ontology
☐	Si1g01090	Nr	gi|514706886|ref|XP_004951168.1	PREDICTED: uncharacterized protein L…	Gbrowse	Indel SNP	Ontology
☐	Si1g01100	Nr	gi|514706882|ref|XP_004951167.1	PREDICTED: protein disulfide isomera…	Gbrowse	Indel SNP	Ontology
☐	Si1g01110	Nr	gi|514720706|ref|XP_004954552.1	PREDICTED: FAM10 family protein At4…	Gbrowse	Indel SNP	Ontology

Total 161844 | 10 /page | 1 2 3 4 5 6 ••• 16185 | Go to 1

Copyright © 2019 | Center for Minor Crop Functional Genomics, Shanxi Agricultural University
No. 1 Ming Xian South Road, Taigu, Shanxi, 030801, P.R. China.

Fig.S3 The annotation page of MDSi.

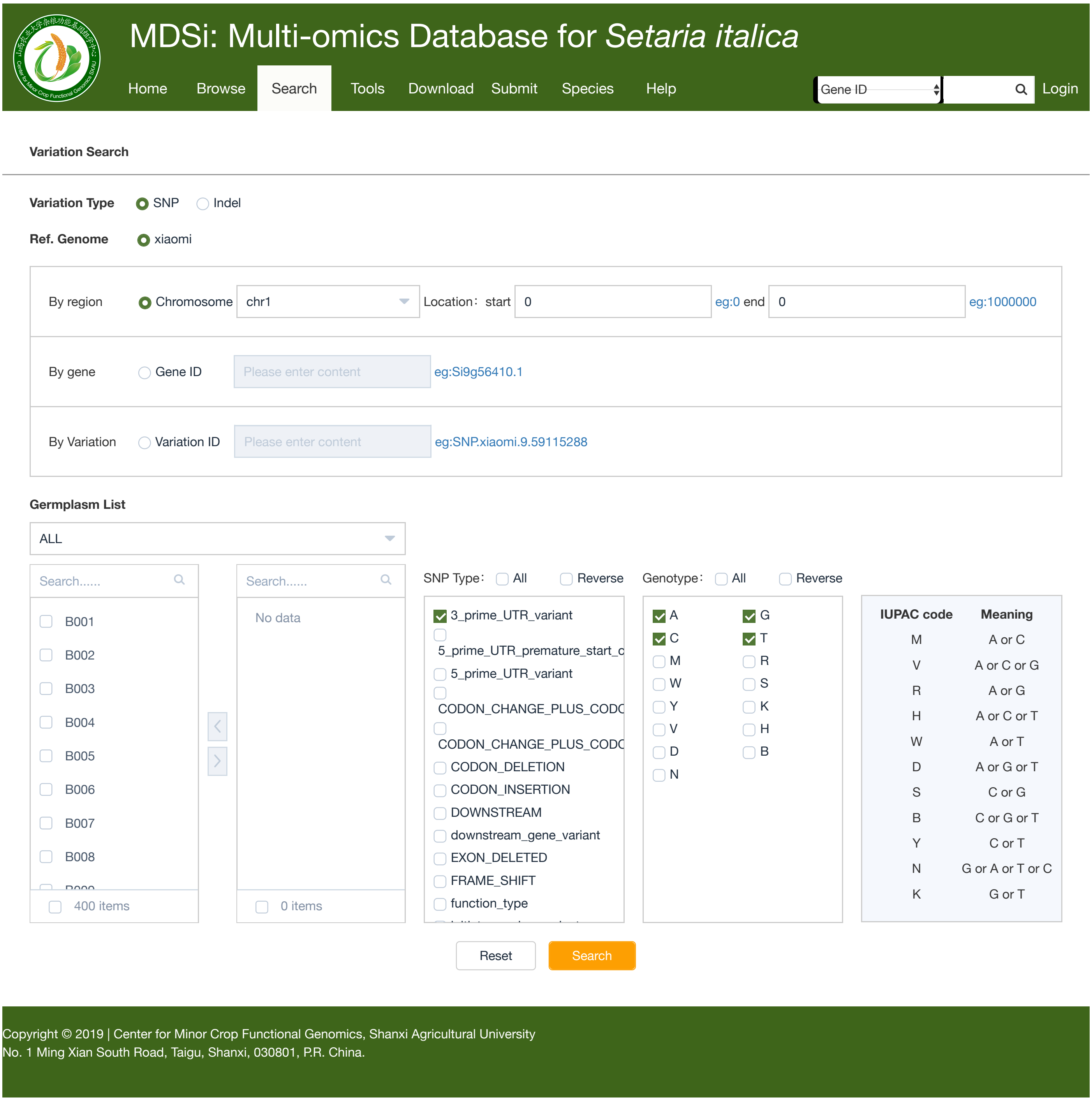

Fig.S4 The variation search page of MDSi.

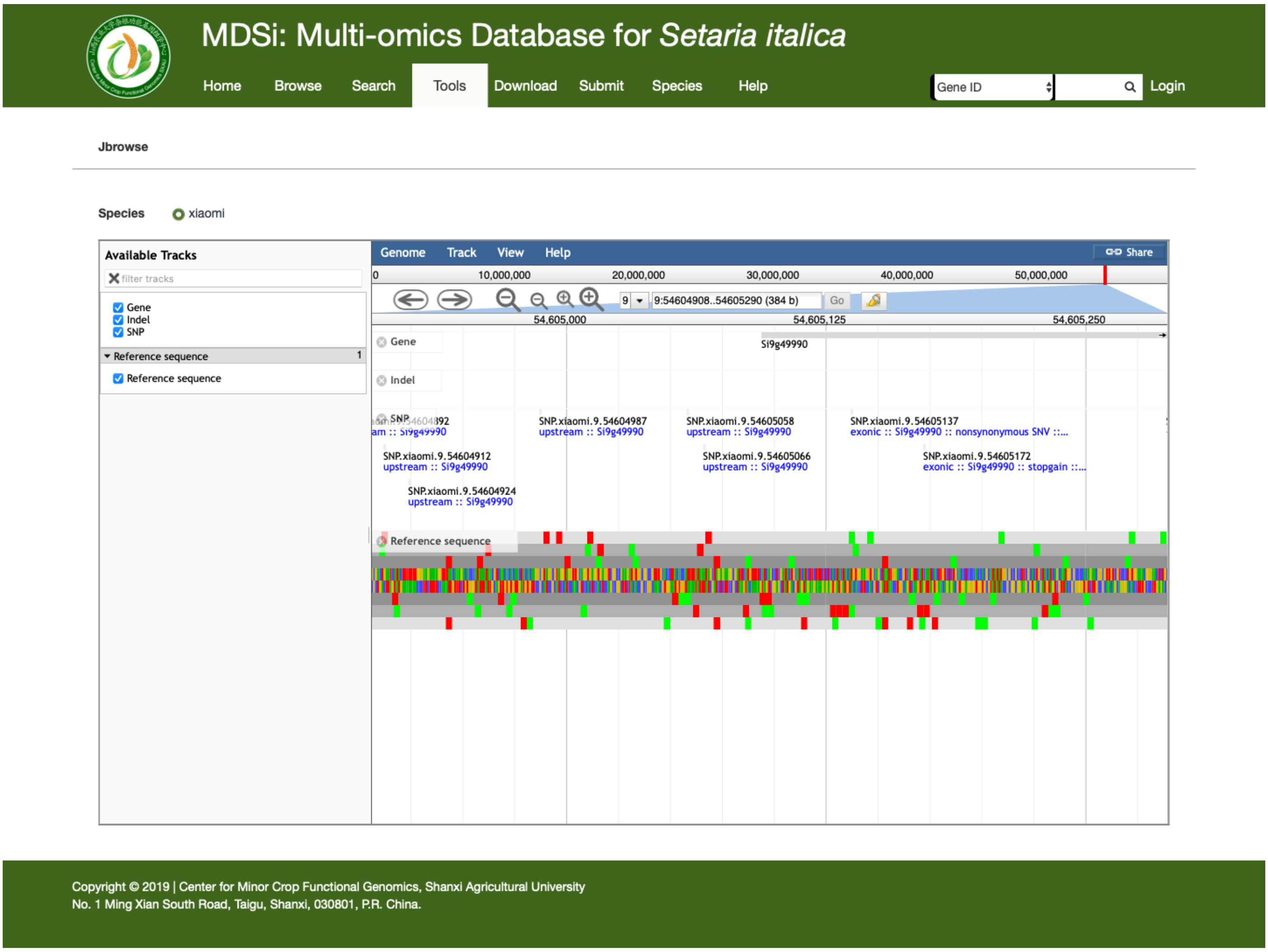

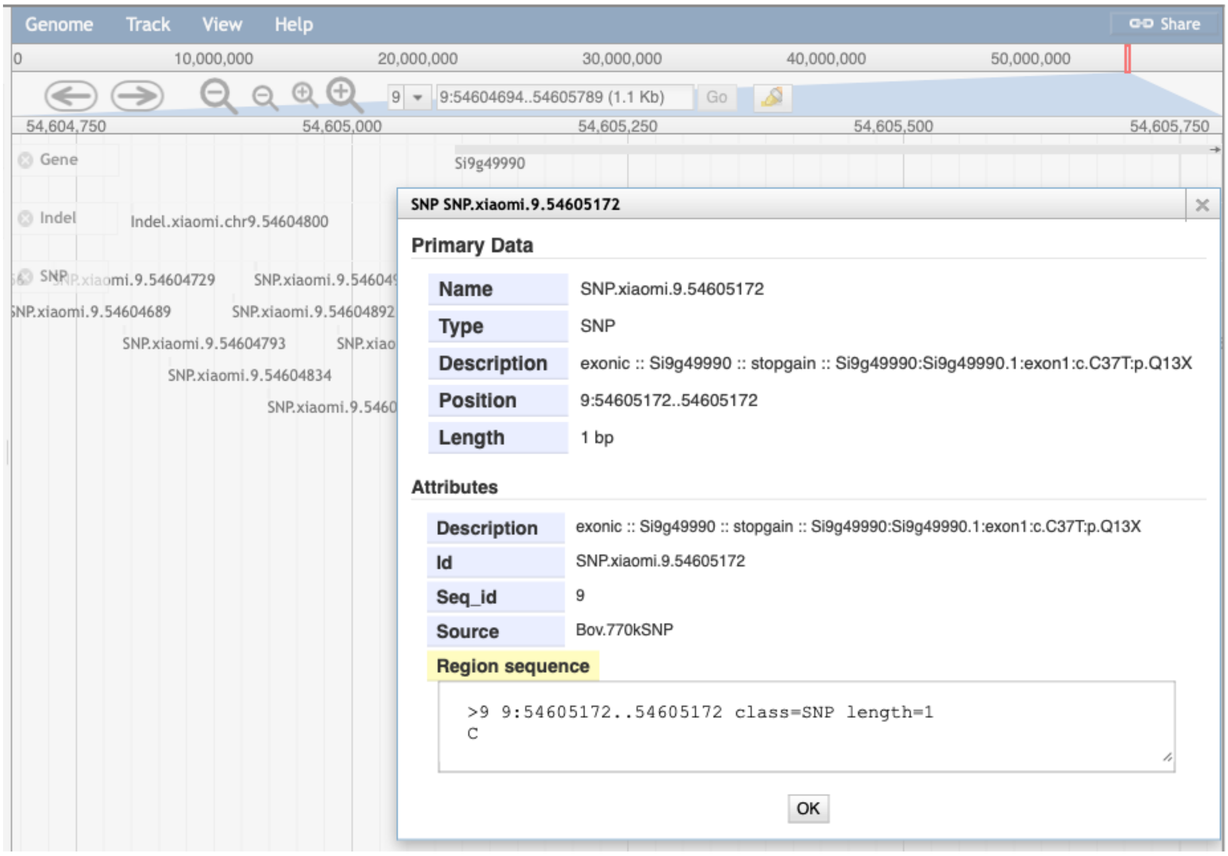

Fig.S5 The JBrowse shows the gene mutations and their impact on *Si9g49990* genes.

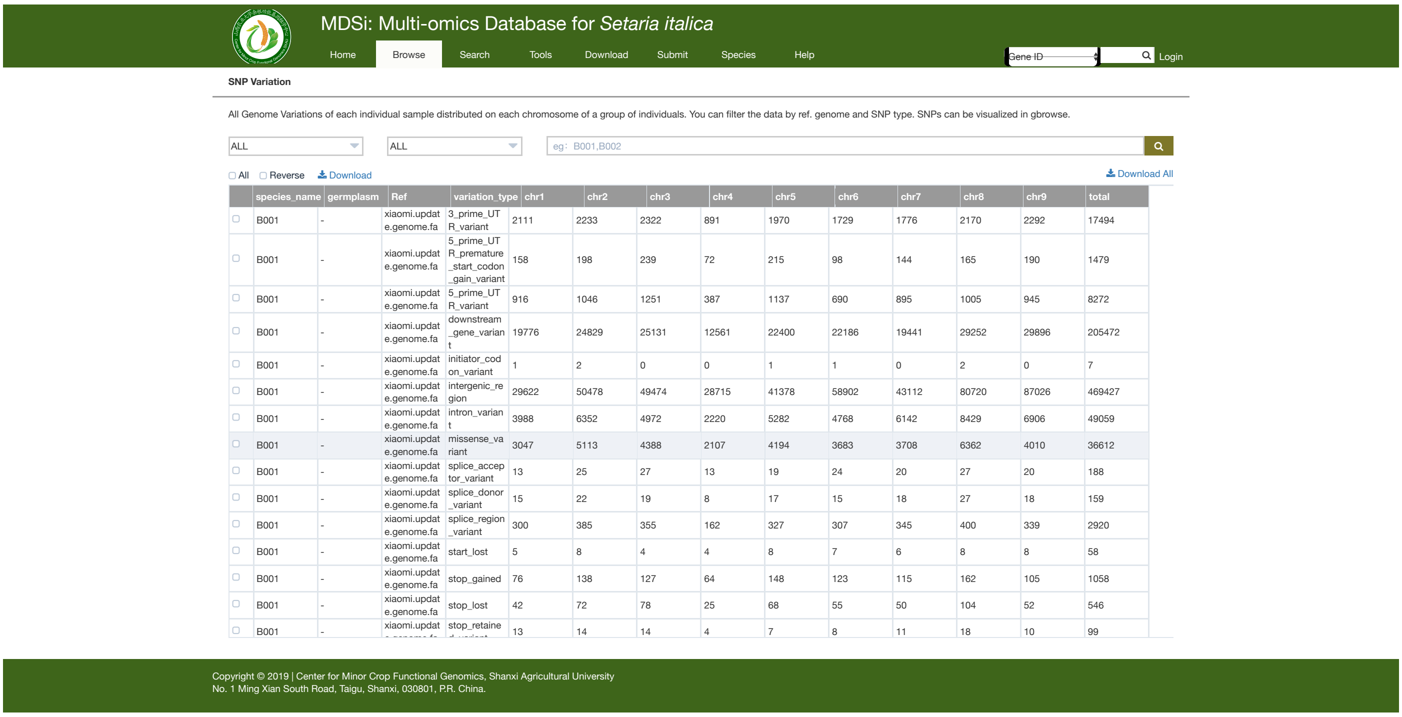

SNP Variation

All Genome Variations of each individual sample distributed on each chromosome of a group of individuals. You can filter the data by ref. genome and SNP type. SNPs can be visualized in gbrowse.

ALL | ALL | eg: B001,B002

All Reverse Download Download All

	species_name	germplasm	Ref	variation_type	chr1	chr2	chr3	chr4	chr5	chr6	chr7	chr8	chr9	total
	B001	-	xiaomi.update.genome.fa	3_prime_UTR_variant	2111	2233	2322	891	1970	1729	1776	2170	2292	17494
	B001	-	xiaomi.update.genome.fa	5_prime_UTR_premature_start_codon_gain_variant	158	198	239	72	215	98	144	165	190	1479
	B001	-	xiaomi.update.genome.fa	5_prime_UTR_variant	916	1046	1251	387	1137	690	895	1005	945	8272
	B001	-	xiaomi.update.genome.fa	downstream_gene_variant	19776	24829	25131	12561	22400	22186	19441	29252	29896	205472
	B001	-	xiaomi.update.genome.fa	initiator_codon_variant	1	2	0	0	1	1	0	2	0	7
	B001	-	xiaomi.update.genome.fa	intergenic_region	29622	50478	49474	28715	41378	58902	43112	80720	87026	469427
	B001	-	xiaomi.update.genome.fa	intron_variant	3988	6352	4972	2220	5282	4768	6142	8429	6906	49059
	B001	-	xiaomi.update.genome.fa	missense_variant	3047	5113	4388	2107	4194	3683	3708	6362	4010	36612
	B001	-	xiaomi.update.genome.fa	splice_acceptor_variant	13	25	27	13	19	24	20	27	20	188
	B001	-	xiaomi.update.genome.fa	splice_donor_variant	15	22	19	8	17	15	18	27	18	159
	B001	-	xiaomi.update.genome.fa	splice_region_variant	300	385	355	162	327	307	345	400	339	2920
	B001	-	xiaomi.update.genome.fa	start_lost	5	8	4	4	8	7	6	8	8	58
	B001	-	xiaomi.update.genome.fa	stop_gained	76	138	127	64	148	123	115	162	105	1058
	B001	-	xiaomi.update.genome.fa	stop_lost	42	72	78	25	68	55	50	104	52	546
	B001	-	xiaomi.updat[illegible]	stop_retaine[illegible]	13	14	14	4	7	8	11	18	10	99

Copyright © 2019 | Center for Minor Crop Functional Genomics, Shanxi Agricultural University
No. 1 Ming Xian South Road, Taigu, Shanxi, 030801, P.R. China.

Fig.S6 The SNP variation page of MDSi.

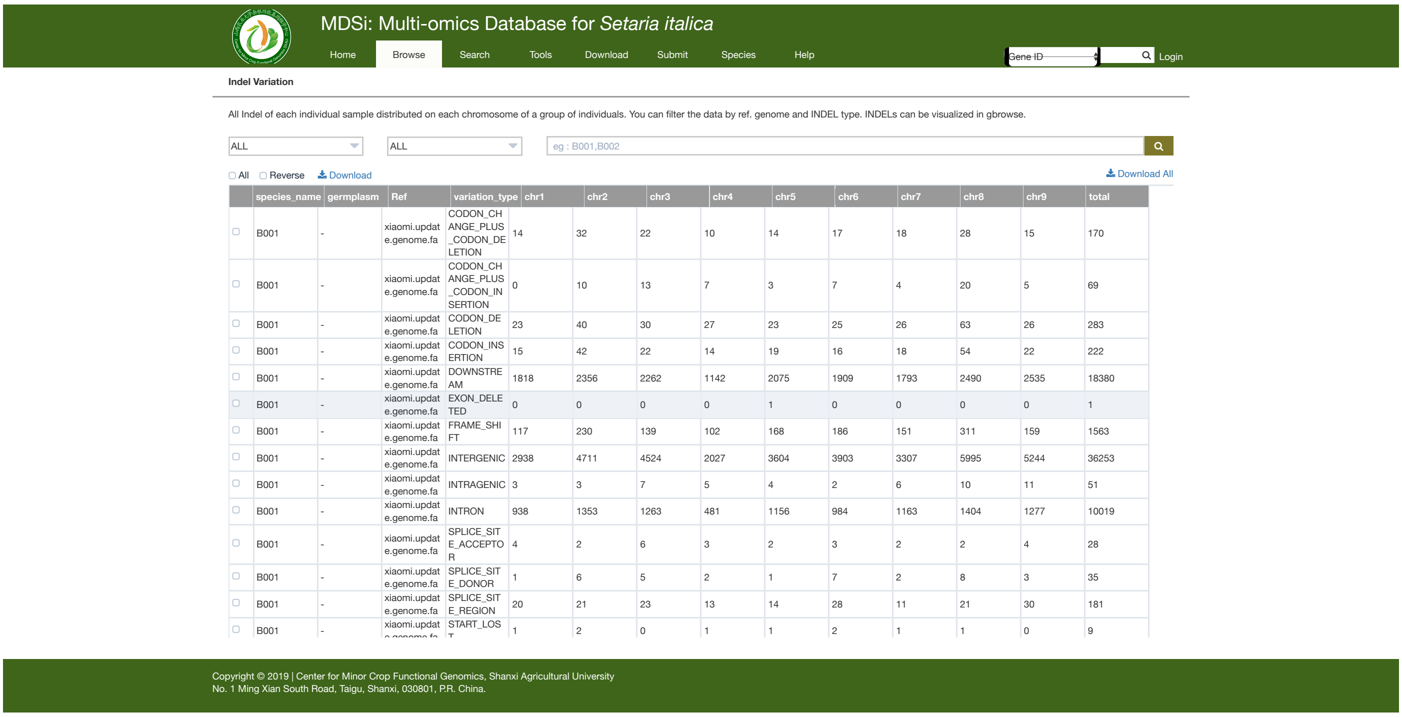

	species_name	germplasm	Ref	variation_type	chr1	chr2	chr3	chr4	chr5	chr6	chr7	chr8	chr9	total
	B001	-	xiaomi.updat e.genome.fa	CODON_CH ANGE_PLUS _CODON_DE LETION	14	32	22	10	14	17	18	28	15	170
	B001	-	xiaomi.updat e.genome.fa	CODON_CH ANGE_PLUS _CODON_IN SERTION	0	10	13	7	3	7	4	20	5	69
	B001	-	xiaomi.updat e.genome.fa	CODON_DE LETION	23	40	30	27	23	25	26	63	26	283
	B001	-	xiaomi.updat e.genome.fa	CODON_INS ERTION	15	42	22	14	19	16	18	54	22	222
	B001	-	xiaomi.updat e.genome.fa	DOWNSTRE AM	1818	2356	2262	1142	2075	1909	1793	2490	2535	18380
	B001	-	xiaomi.updat e.genome.fa	EXON_DELE TED	0	0	0	0	1	0	0	0	0	1
	B001	-	xiaomi.updat e.genome.fa	FRAME_SHI FT	117	230	139	102	168	186	151	311	159	1563
	B001	-	xiaomi.updat e.genome.fa	INTERGENIC	2938	4711	4524	2027	3604	3903	3307	5995	5244	36253
	B001	-	xiaomi.updat e.genome.fa	INTRAGENIC	3	3	7	5	4	2	6	10	11	51
	B001	-	xiaomi.updat e.genome.fa	INTRON	938	1353	1263	481	1156	984	1163	1404	1277	10019
	B001	-	xiaomi.updat e.genome.fa	SPLICE_SIT E_ACCEPTO R	4	2	6	3	2	3	2	2	4	28
	B001	-	xiaomi.updat e.genome.fa	SPLICE_SIT E_DONOR	1	6	5	2	1	7	2	8	3	35
	B001	-	xiaomi.updat e.genome.fa	SPLICE_SIT E_REGION	20	21	23	13	14	28	11	21	30	181
	B001	-	xiaomi.updat [illegible]	START_LOS [illegible]	1	2	0	1	1	2	1	1	0	9

Fig.S7 The InDel variation page of MDSi.

Gene network

Gene networks characterize the interactions of bio-molecules such as the physical interactions, metabolite flow, regulatory relationships, co-expression relationships, and more. Network analysis can be used to identify related genes in the same biological processes or pathways etc. The foxtail millet gene network (FGN) was constructed using the context likelihood of relatedness (CLR) algorithm (mutual information method, an extension of the relevance networks based on RNA-Seq expression data, which means that the connected genes in the FGN have similar expression profile. The CLR network with a Z-score threshold 3.49 was determined as the current FGN. The larger of a Z-score between two genes indicated the more reliable interaction (more similar expression profile). You can search genes which have similar expression profiles through the bellow two search boxes.

Total : 21

Gene : Si1g01230

SVG Download

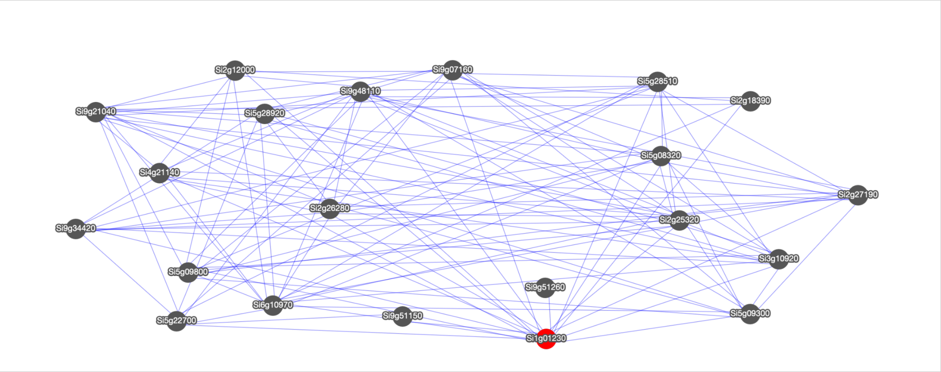

Download All

Target gene	Description	P-values
Si5g09800	transferase activity, transferring acyl groups other than amino-acyl groups; K13065 shikimate O-hydroxycinnamoyltransferase [EC:2.3.1.133] ; PREDICTED: shikimate O-hydroxycinnamoyltransferase-like [Setaria italica]	0.999029387631966
Si5g08320	borate transmembrane transport; cytoplasmic membrane-bounded vesicle; borate transmembrane transporter activity; purine nucleobase transport; integral component of membrane; water channel activity; K09874 aquaporin NIP ; PREDICTED: aquaporin NIP4-1-like [Setaria italica]	0.998222782302236
Si5g09300	dihydrokaempferol 4-reductase activity; oxidation-reduction process; cellular metabolic process; coenzyme binding; PREDICTED: tetraketide alpha-pyrone reductase 2-like [Setaria italica]	0.999567345568502
Si5g28510	cytoplasmic membrane-bounded vesicle; PREDICTED: probable non-specific lipid-transfer protein-like [Setaria italica]	0.998589276904005
Si5g28920	transmembrane transport; integral component of membrane; ATPase activity, coupled to transmembrane movement of substances; obsolete ATP catabolic process; ATP binding; K05658 ATP-binding cassette, subfamily B (MDR/TAP), member 1 [EC:3.6.3.44] ; PREDICTED: ABC transporter B family member 11-like [Setaria italica]	0.999738251088838
Si9g34420	alcohol metabolic process; flavin adenine dinucleotide binding; cytoplasmic membrane-bounded vesicle; lyase activity; choline dehydrogenase activity; oxidation-reduction process; PREDICTED: protein HOTHEAD-like [Setaria italica]	0.999364469010184
Si9g51150	cytoplasmic membrane-bounded vesicle; electron carrier activity; iron ion binding; heme binding; oxygen binding; alkane 1-monooxygenase activity; sporopollenin biosynthetic process; aromatase activity; electron transport chain; K20495 long-chain fatty acid omega-monooxygenase [EC:1.14.13.205] ; PREDICTED: cytochrome P450 704C1-like isoform X2 [Setaria italica]	0.998881951546037
Si3g10920	cytoplasmic membrane-bounded vesicle; electron carrier activity; monooxygenase activity; iron ion binding; heme binding; oxidoreductase activity, acting on paired donors, with incorporation or reduction of molecular oxygen; electron transport chain; PREDICTED: cytochrome P450 86B1-like [Setaria italica]	0.998088694954223
Si9g51260	pollen exine formation; fatty-acyl-CoA reductase (alcohol-forming) activity; plastid; K13356 alcohol-forming fatty acyl-CoA reductase [EC:1.2.1.84] ; PREDICTED: fatty acyl-CoA reductase 2-like [Setaria italica]	0.997652246349817
Si2g25320	exine; PREDICTED: BURP domain-containing protein 15-like [Setaria italica]	0.999712748029476
Si5g22700	cytoplasmic membrane-bounded vesicle; PREDICTED: uncharacterized protein LOC101782860 [Setaria italica]	0.998466074833659
Si2g26280	coenzyme binding; endoplasmic reticulum; catalytic activity; sporopollenin biosynthetic process; seed development; nucleotide binding; PREDICTED: tetraketide alpha-pyrone reductase 1-like [Setaria italica]	0.999940078228995
Si2g27190	transition metal ion binding; acyl-[acyl-carrier-protein] desaturase activity; fatty acid metabolic process; membrane; plastid; oxidation-reduction process; K03921 acyl-[acyl-carrier-protein] desaturase [EC:1.14.19.2 1.14.19.11 1.14.19.26] ; PREDICTED: acyl-[acyl-carrier-protein] desaturase 4, chloroplastic-like [Setaria italica]	0.999476960592985
Si2g18390	PREDICTED: BURP domain-containing protein 14-like [Setaria italica]	0.99908467480145
Si2g12000	tetraketide alpha-pyrone synthase activity; phenylpropanoid biosynthetic process; polyketide biosynthetic process; endoplasmic reticulum; sporopollenin biosynthetic process; PREDICTED: chalcone synthase 1-like [Setaria italica]	0.998179901565073
Si6g10970	PREDICTED: uncharacterized protein LOC101773562 [Setaria italica]	0.997780603488466
Si4g21140	ATPase activity, coupled to transmembrane movement of substances; obsolete ATP catabolic process; pollen exine formation; ATP binding; pollen maturation; plasma membrane; PREDICTED: ABC transporter G family member 26-like [Setaria italica]	0.999833930369887
Si9g07160	cytoplasmic membrane-bounded vesicle; PREDICTED: actin cytoskeleton-regulatory complex protein PAN1-like [Setaria italica]	0.997891165165816
Si9g48110	transferase activity, transferring hexosyl groups; metabolic process; PREDICTED: UDP-glucose flavonoid 3-O-glucosyltransferase 7-like [Setaria italica]	0.999576600172999
Si9g21040	transferase activity, transferring acyl groups other than amino-acyl groups; pollen exine formation; biosynthetic process; PREDICTED: stilbene synthase 4-like [Setaria italica]	0.999330530251223

Copyright © 2019 | Center for Minor Crop Functional Genomics, Shanxi Agricultural University
No. 1 Ming Xian South Road, Taigu, Shanxi, 030801, P.R. China.

Fig.S8 The network result page of *Si1g01230* gene.

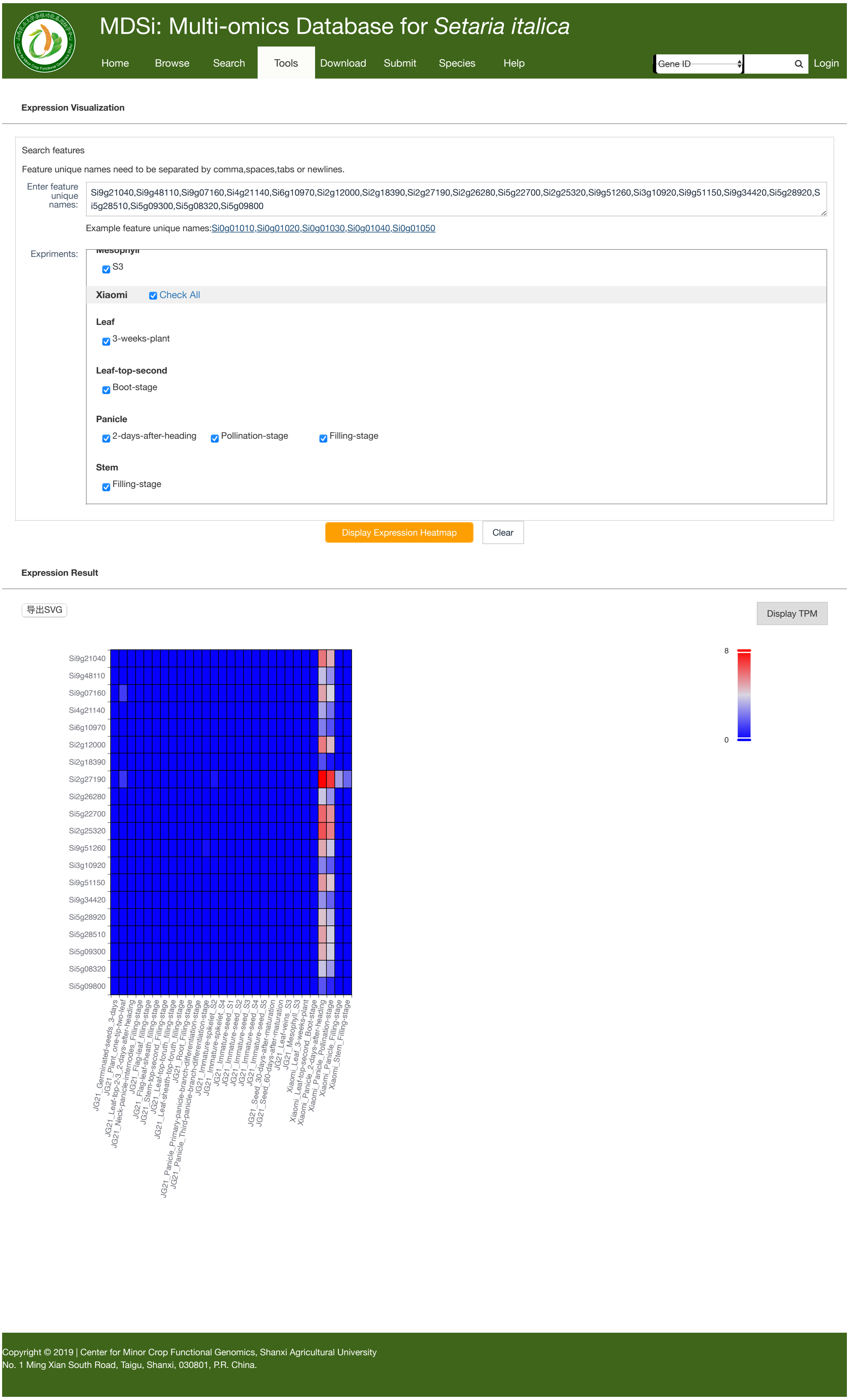

Fig.S9 The network result of *Si1g01230*.

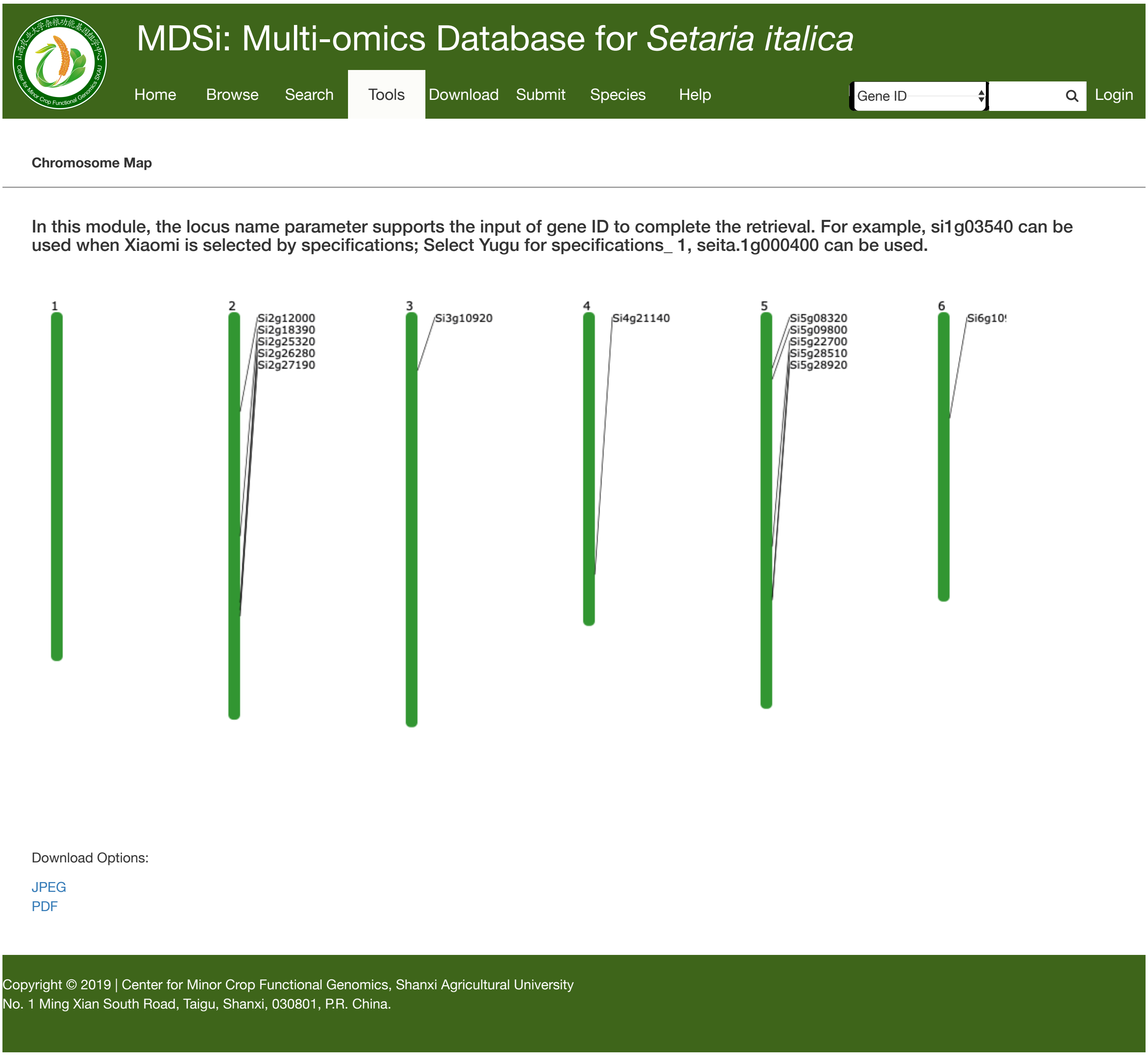

Fig.S10 The chromosome location of genes related to Si1g01230.

MDSi: Multi-omics Database for *Setaria italica*

Home Browse Search Tools Download Submit Species Help Gene ID Login

Download

这个地方可以为Download模块，增加描述信息-2021年8月31号测试

Germplasm material

A total of 398 resources are included in this database. The information of some resources and their corresponding numbers are shown in the table below.
Click **More** to view the details of 398 resources.

Sample ID	中文名	Name	来源	Latitude (N)	Longitude (E)	Type	Structure	NCBI Accession	GSA Accession
B001	泡子沿谷子-1	Paoziyanguzi-1	黑龙江省爱辉	50.252	127.501	Chinese landrace	North_landrace	SRR11804962	CRX110418
B002	老来变	Laolaibian	黑龙江省孙吴清溪	49.330	127.139	Chinese landrace	North_landrace	SRR11804961	CRX110419
B003	大糟皮	Dacaopi	黑龙江嫩江县	49.166	125.214	Chinese landrace	North_landrace	SRR11804946	CRX110420
B004	糟皮一刀把齐	Caopi-daobaqi	黑龙江嫩江县	49.166	125.214	Chinese landrace	North_landrace	SRR11805249	CRX110421
B005	鸭子嘴	Yazizui	黑龙江克山县	48.032	125.865	Chinese landrace	North_landrace	SRR11804870	CRX110422
B006	佛顶珠	Fodingzhu	黑龙江拜泉县	47.607	126.085	Chinese landrace	North_landrace	SRR11805021	CRX110423
B007	红粘谷	Hongniangu	黑龙江德都县	48.511	126.186	Chinese landrace	North_landrace	SRR11805198	CRX110424
B008	刀把齐	Daobaqi	黑龙江嫩江县	49.166	125.214	Chinese landrace	North_landrace	SRR11805187	CRX110425

- Xiaomi annotation V1.0

- Genome_sequence.fa.zip
- Genes.zip
- Peptids.zip
- Transcription.zip
- CDS.zip

Copyright © 2019 | Center for Minor Crop Functional Genomics, Shanxi Agricultural University
No. 1 Ming Xian South Road, Taigu, Shanxi, 030801, P.R. China.

Fig.S11 The download page of MDSi.
